# How do children travel to school in urban India? A cross-sectional study of 5,842 children in Hyderabad

**DOI:** 10.1186/s12889-016-3750-1

**Published:** 2016-10-19

**Authors:** Shailaja Tetali, P. Edwards, G. V. S. Murthy I. Roberts

**Affiliations:** 1 Indian Institute of Public Health-Hyderabad, Public Health Foundation of India, ANV Arcade, Plot No.1, Amar Cooperative Society, Kavuri Hills, Madhapur, Hyderabad 500033 India; 2 Department of Population Health, London School of Hygiene & Tropical Medicine, Keppel Street, London, WC1E 7HT UK

**Keywords:** Walking, Cycling, Children, Travel, School, India

## Abstract

**Background:**

Millions of children travel to school every day in India, yet little is known about this journey. We examined the distribution and determinants of school travel in Hyderabad, India.

**Methods:**

We conducted a cross-sectional survey using a two-stage stratified cluster sampling design. School travel questionnaires were used to collect data from children aged 11–14 years, attending private, semi-private and government funded schools in Hyderabad. We used Google Earth to estimate the distance from home to school for each child and modelled the relationship between distance to school and mode of travel, adjusting for confounders.

**Results:**

Forty five of the 48 eligible schools that were selected agreed to participate, providing a total sample of 5842 children. The response rate was 99 %. Most children walked (57 %) or cycled (6 %) to school but 36 % used motorised transport (mostly bus). The proportion using motorised transport was higher in children attending private schools (41 %) than in those attending government schools (24 %). Most (90 %) children lived within 5km of school and 36 % lived within 1km. Greater distance to school was strongly associated with the use of motorised transport. Children living close to school were much more likely to walk or cycle.

**Conclusions:**

Most children in Hyderabad walk (57 %) or cycle (6 %) to school. If these levels are to be maintained, there is an urgent need to ensure that walking and cycling are safe and pleasant. Social policies that decrease distances to school could have a large impact on road traffic injuries, air pollution, and physical activity levels.

## Background

India, the second most populous country in the world, is rapidly motorising. The number of registered motor vehicles in India is increasing by over 12 % per year [1]. There were 112 million registered motor vehicles on India’s roads in 2010 and by 2030 there could be 500 to 600 million vehicles [2]. This enormous increase in motor vehicle use will have important implications for air quality, road traffic injuries, physical activity and climate change.

Although millions of children travel to school every day in India, [3] relatively little is known about their journeys. However, escorting children to school is known to account for a large proportion of household travel, and in most cities, peak traffic density coincides with the beginning and the end of the school day [4]. Given the number of school related trips in India, the choice of transportation modes used is likely to have major public health implications.

Studies in high income countries show that distance to school is one of the most important determinants of transportation mode. The prevalence of walking and cycling decreases and the use of motorised travel increases with increasing distance to school [5–8]. Other factors associated with motor vehicle use are young age, [9–11] female gender, [12, 13] parental concerns about safety, [8, 14] physical infrastructure, and weather conditions [6]. Information on travel to school in rapidly developing Indian cities is needed to inform public policy decisions in education, transport and public health.

This study examines travel to school in Hyderabad, the fifth largest city in India with a population, employment mix and transport network that is comparable to other large Indian metropolitan cities.

## Methods

### Survey design

We conducted a cross-sectional survey using a two-stage stratified cluster sampling design. The strata were geographical (16 mandals, equivalent to boroughs) and administrative (types of school management).

There are three main types of schools in Hyderabad: government, semi-private and private schools. ‘Government’ schools are run by the Central or State Government; ‘semi-private’ schools are government-aided schools which are managed privately but receive regular maintenance grant from the government, local body or any other public authority; and ‘private’ schools which are run by a Society or a Trust without government aid [15]. There are 802 government schools, 342 semi-private schools, and 1,899 private schools in Hyderabad. We considered type of school to be a marker of socio-economic status and parental influence: generally, government schools cater to lower income families, semi-private schools cater to middle income families and children from higher income families attend private schools.

### Participants

We obtained lists of all schools in each mandal in Hyderabad district with grades 6–9 (typically children aged 11–14 years) from the District Education Office. We selected one school of each type from each mandal at random, using random numbers generated using the software R. In each school selected the principal randomly selected two sections (i.e. classrooms which normally have 30–40 children) in grades 6–9. Where schools had only one section in grades 6–9, it was selected. All children in grades 6–9 who were present on the day of the survey were included in the study. Assuming that the true prevalence of walking to school was 50 % [16], we estimated that a sample of 6,000 children would be required to be 95 % confident that the sample estimate would be within 5 % of the true prevalence.

### Questionnaire

We prepared a self-completion questionnaire with 21 questions about distance and mode of travel to school and conducted extensive piloting of the questionnaire [17]. The questionnaire collected information on the usual mode of travel to school, mode of travel during wet or dry weather conditions, parental permissions for independent travel, children’s perception of safety, and physical activity after school. We used an English version of the questionnaire in private schools, and a Telugu version (which was the language of instruction) in government and semi-private schools. The questionnaire was administered during a regular class period and could be completed in 15–20 min.

### Variables

The outcome variable was children’s usual mode of travel to school. The exposure variable was distance to school. Potential confounding variables were grade, gender, school type, physical activity, and parental permissions for independent mobility. We estimated distance from home to school using Google Earth^TM^ based on the school location and self-reported nearest landmark to home. The estimated distance has been shown to be accurate to within 65m (-30m to 159m) for walking and cycling and to within 325m (-664m to 1314m) for motorised transport [17].

Modes of transport were categorised as *walking, cycling, auto-rickshaw* and *cycle rickshaw* (commercial three-wheeled passenger vehicles)*, school bus* (private)*, RTC bus* (public road transport corporation bus)*, motorised two-wheeler* (motorbike), *car* and *train*. We assessed independent mobility by asking whether children were allowed to cycle and to cross main roads on their own. Distance to school was categorised as: 0.25 to 0.5km; 0.5 to 0.75km; 0.75 to 1km; 1.0 to 1.25 km; 1.25 to 1.5km; 1.5 to 2km; 2 to 2.5km; 2.5 to 3km; 3 to 5km and >5 km. These distance categories were chosen to ensure similar sample sizes in each group. Grades were categorised as grade 6, 7, 8 or 9. Physical activity was categorised as the number of days and hours exercised after school during the past week.

### Data collection

Research assistants with survey and interview experience conducted the survey in the schools, in the presence of the class teachers. The survey was conducted from November 2013 to February 2014. Each question was read out aloud by a study investigator, allowing plenty of time for the children to give their responses. Only after all children in a class had answered one question did the study investigator read out the next question, until all questions had been answered. This ensured that any questions, or doubts, that children had were attended to immediately, so no child would feel left out. The study investigator made monitoring visits to schools to ensure that each question was read out and explained to the children.

### Probability weights

For each stratum, we estimated the probability of each school being selected (first stage of sampling), followed by the probability of each section being selected (second stage). The probability of selection at the first stage was the reciprocal of the number of schools in each stratum. The probability of selection at the second stage was the number of sections of each grade selected by principals, divided by the number of sections of each grade in each school (which was recorded when principals selected the sections). We checked the probability weights by comparing the population size estimated when applying the weights, with the numbers of children in grades 6–9 in each mandal recorded in state education department reports [18, 19].

### Statistical analysis

We examined associations between travel mode and distance to school, stratified by school type. We used logistic regression to estimate odds ratios with 95 % confidence intervals for the association between walking and cycling and distance to school, adjusting for potential confounding factors (e.g. grade, gender, school type, independent mobility, physical activity). We used the ‘survey’ commands in Stata to account for stratification, clustering and unequal probability of selection, and the ‘test’ command to test the associations in the logistic regression models. We retained variables that remained statistically significant at the 5 % level in the ‘best fit’ model. We analysed data using STATA/SE V.12.0 (Stata Corporation, Texas, USA).

## Results

### Sample characteristics

Forty five of the 48 eligible schools that were selected agreed to participate, providing a total sample of 5842 children (Table 1). Three schools refused due to time constraints. Three percent of children in the participating schools were absent on the day of the survey (*n* = 179). Compared to those present, absentees had similar age (12.9 vs 13.1 years), and sex (44 % vs 47 % boys), and prevalence of walking (74 % vs 69 %). Almost all children (99 %) provided a valid home address, or nearest landmark, for the estimation of distance to school. Forty children did not answer the question on mode of travel, and 76 children did not provide the information on the nearest landmark. The mean age of the children in the sample was 13 years (SD 1.3 years). There was a higher proportion of girls (54 %) in the sample.

**Table 1:** Characteristics of the sample

	Government	Semi-private	Private	Total
Number of schools	16	15	14	45
n (%)	1,836 (31)	1,585 (27)	2,421 (41)	5,842 (100)
Boys n (%)	768 (42)	762 (48)	1,129 (47)	2,659 (46)
Girls n (%)	1,068 (58)	823 (52)	1,292 (53)	3,183 (54)
Age in years (mean, SD)	13 (2)	13 (2)	13 (1)	13 (1.3)

### Main results

#### Mode of travel

All the children surveyed were capable of walking or cycling to school. Most children walked (57 %) or cycled (6 %) to school but 36 % used motorised transport (mostly bus). Greater distance to school was strongly associated with the use of motorised transport. Sixty-four children responded that they walked as well as travelled by RTC (public transport) bus and were assigned to the category ‘RTC bus.’

#### Distance to school

The average distance to school was 2 km (SD 2.6 km). Most children (90 %) lived within 5km of school, many (69 %) lived within 2 km, and about a third (36 %) lived within 1km.

#### Relationship between distance and walking or cycling

**Fig. 1:**
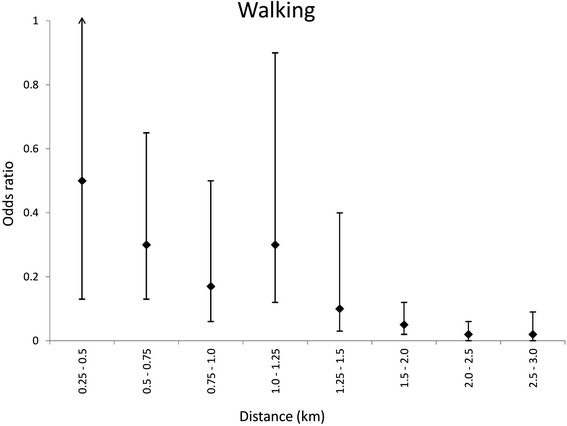
Relationship between distance and walking to school. Odds ratios adjusted for gender, grade, type of school, mode of travel, hours of exercise and travel alone

**Fig. 2:**
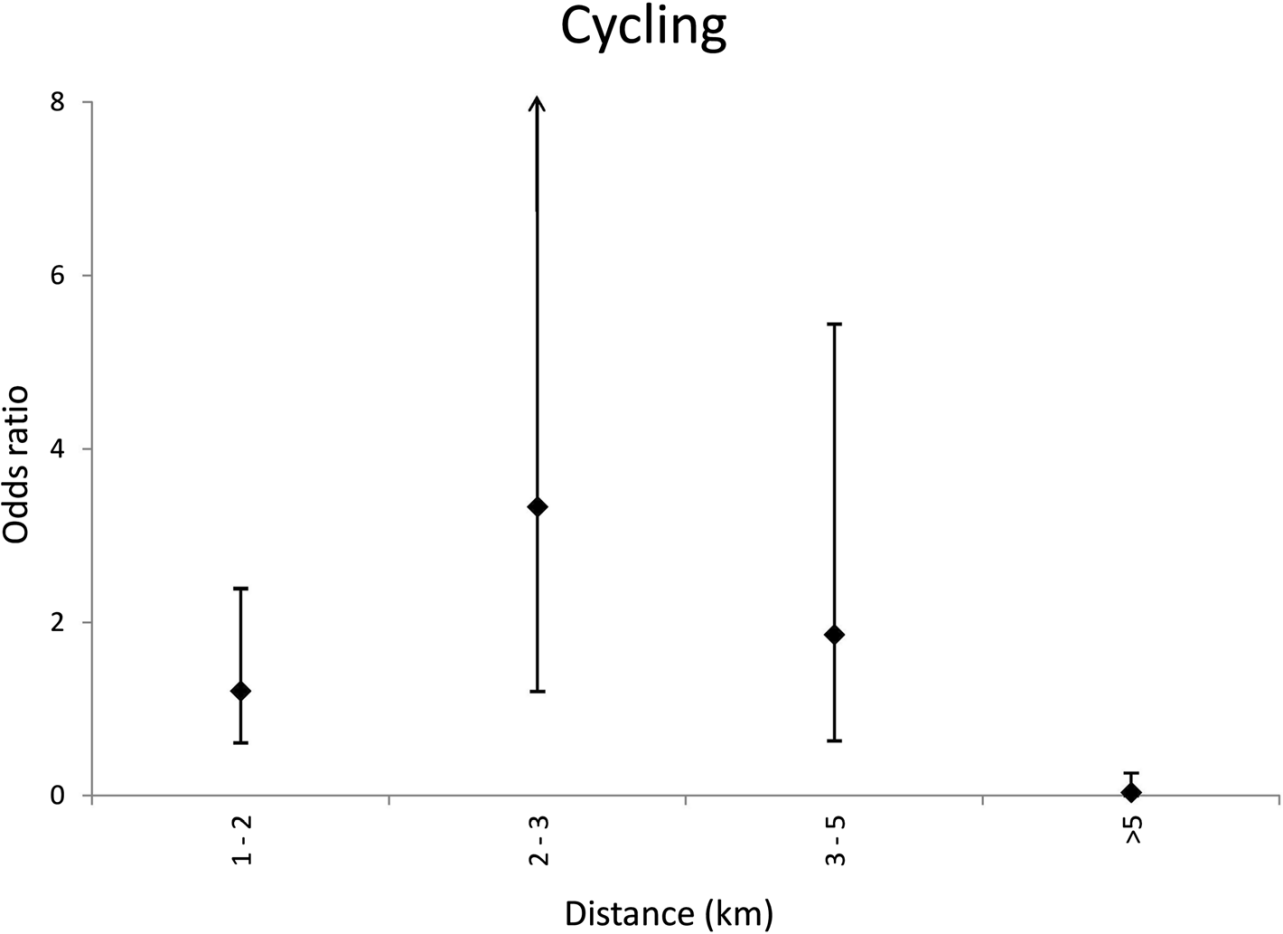
Relationship between distance and cycling to school. Odds ratios adjusted for gender, grade, type of school, mode of travel, hours of exercise and travel alone

Walking to school was inversely associated with distance. Compared to children living within 0.25km of school (baseline group), children living 0.25–0.5km from school were half as likely (OR = 0.5) to walk to school, and children living 0.5–0.75km from school were around 70 % less likely (OR = 0.3) to walk to school (Fig. 1). Compared to children living within 1km of school (baseline group), children living 2–3km from school were over three times as likely to cycle to school (OR = 3.3) (Fig. 2).

#### Other factors associated with walking and cycling

Children in the 8^th^ grade were twice as likely to cycle as those in the 6^th^ grade (OR 2.5; 95 % confidence interval 1.4 to 4.2). ) Girls were less likely to cycle (OR 0.15; 95 % CI 0.07 to 0.3) than boys. Children who travelled to school alone were approximately three times more likely to walk or cycle to school, compared to those who were accompanied (OR 3.3; 95 % CI 2.3 to 4.6) Similarly, children who reported exercising after school were more likely to walk to school than those who did not exercise. Children who exercised for 7 h a week were almost twice as likely to cycle to school as children who got no exercise (OR 1.9; 95 % CI 0.92 to 4.1).

#### Mode of travel by type of school

A higher proportion of children in government schools walked (69 %) compared with those in private schools (53 %) (Table 2). Prevalence of cycling was similar (6 %) across school types. The proportion using motorised transport was higher in children attending private schools (41 %) than in those attending government schools (24 %). RTC bus use was more common in children attending government schools than in private schools (19 % versus 2 %). Children attending private schools also travelled 0.9 km further, on average, than their counterparts attending semi-private schools.

**Table 2:** Distribution of usual mode of travel to school by type (adjusted for survey design)

Travel mode to school	Government		Semi-private		Private		Overall	
	%	(95 % CI)	%	(95 % CI)	%	(95 % CI)	%	(95 % CI)
Walk	69.0	(58, 79)	68.0	(59, 76)	53.0	(34, 71)	57.0	(41, 71)
Cycle	6.0	(4, 11)	6.0	(4, 9)	6.0	(3, 9)	6.0	(4, 8)
School bus	0.6	(0.2, 2)	1.0	(0.2, 8)	11.0	(5, 21)	8.0	(4, 17)
Car	0.5	(0.2, 1)	0.2	(0, 1)	5.0	(2, 16)	4.0	(1, 12)
2 wheeler	2.0	(1 , 3)	10.0	(6, 16)	11.0	(7, 16)	9.0	(6, 14)
RTC bus	19.0	(10, 34)	10.0	(4 , 25)	2.0	(1, 5)	5.0	(3, 10)
Auto-rickshaw	2.0	(1, 6)	4.0	(2, 7)	12.0	(5, 27)	10.0	(4, 21)
Cycle-rickshaw	1.0	(0, 1)	1.0	(0.2, 1)	0.3	(0.1, 1)	0.3	(0.1, 0.5)
Train	0.0	(0, 0)	0.0	(0, 0.3)	0.0	(0, 0)	0.0	(0, 0)
Other	0.1	(0, 1)	0.1	(0, 1)	1.0	(0.3, 3)	0.07	(0.3, 2)
Distance (km) to school (mean, SD)	1.7	(2.4)	1.4	(2.9)	2.3	(2.1)	2.0	(2.6)

## Discussion

This study found that most children in Hyderabad (57 %) walk or cycle (6 %) to school. Distance to school was strongly associated with the use of motorised transport. Children attending private schools travelled almost 1km further and were more likely to travel by car (5 %) instead of those attending semi-private schools (0.2 %). Compared to children living within 1km of school, children living 2–3km from school were over three times as likely to cycle to school.

### Limitations of this study

Our estimates of children’s usual mode of travel to school are based on self-reports, which are susceptible to information bias. Children who were absent on the day of the survey were not included in the survey. They might well be different; however, they were very few. We used information based on children’s home address and nearest landmark, to estimate the distance to school. The landmark based method showed minimal evidence of bias and gave reasonably accurate estimates of distance to school. It is found to be a feasible method, in the absence of GPS equipment and software, especially in low resource urban settings [17]. We were not able to select classrooms, which were selected by school principals, based on the availability of a free period for children to complete the survey. This could introduce bias if the principal selected the most literate or physically active children, but this is unlikely because classrooms are generally balanced for good, average, or moderate performers. Therefore the probability of any child being in the survey should be the same. Forty children did not provide their mode of travel, and 76 children did not give a valid address. These children were excluded from the analysis and this may have biased our results. We did not collect information on religion which is another potentially confounding variable.

Despite these limitations, this is the first study of children’s commuting to school in India. We achieved a 99 % response rate from children attending private, semi-private and government schools. The large sample size and high response rate are important strengths. We used a questionnaire that had been shown to be valid and reliable, (which confirmed that children were capable of answering questionnaires by themselves). The question on usual mode of travel showed ‘almost perfect’ agreement using the kappa statistic during reliability testing. We estimated distance to school based on children’s home address and landmark. Because our method was accurate to within 65m (-30m to 159m) of the true distance, [17] we are reasonably confident in the results of the relationship between distance and walking/cycling to school.

We used a stratified clustered sampling design to ensure that the sample included government, semi-private and private schools in each of the geographical boroughs of Hyderabad. We used survey commands in Stata for analysis to adjust for probability of selection, stratification and clustering. We estimate that our random sample of 5,842 children is representative of the target population of 322,258 children in Hyderabad. Our results might therefore be generalised to children aged 11–14 in other urban areas in India, with similar population sizes and transport networks as Hyderabad.

### Comparisons with other studies

Distance to school has a strong effect on mode choice [5, 20]. Two-thirds of the children in our study lived within a mile from school, and overall, most (63 %) walked or cycled. In comparison, a fifth of the children lived within a mile from school in the USA and overall, 12 % walked or cycled [21].

As shown in high income settings, boys were more likely to cycle to school than girls and older children were more likely to cycle than younger children [12, 22]. These findings reflect cross-cultural social norms related to children’s independent travel.

Walking was more common in government and semi-private schools than in private schools. The Indian government provides free education but it does not pay for transportation. Children in lower income families walk if they cannot afford bicycles. Children in higher income families have greater access to motor vehicles and we found that a greater proportion of children at private schools travel by motorised transport. The type of school in India is an indicator of socio-economic status. Similarly, a British study found attendance at an independent school to be a strong predictor of car travel [14]. We also found that children who exercised after school hours were also more likely to walk to school.

The prevalence of active commuting of 63 % in our sample is higher than in countries which have pavements and cycle lanes. Although commuting by car is currently available to only 4 % of children in Hyderabad, it is likely to increase, given the 12 % annual growth of motor vehicles in India. India can avoid the mistakes of other motorised countries and could mitigate unintended consequences like road traffic injuries [23]. Infrastructure such as pavements for walking and safe space for cycling need to be improved, to preserve independent travel and increase children’s physical activity.

### Meaning of the study and future research

There is evidence to suggest that everyday travel by walking and cycling is associated with positive health benefits for children [24, 25]. School journeys provide this opportunity to walk and cycle, with the associated public health impacts of these journeys. The relationship between distance and mode presented in this study is new information, especially among children in urban India.

Compared to children in the UK and USA, most children in India walk or cycle to school. This is in spite of few pavements and cycle lanes [26]. The reasons for mode choice including barriers to walking and cycling, and the extent of parental influence will be useful to explore through future research. Ensuring that walking and cycling are safe, enjoyable and convenient modes of urban transport for short journeys is critical for improving health and ensuring ecological sustainability [27]. This study contributes to understanding children’s school travel in Hyderabad, which is a crucial first step for drawing attention to an area which has so far been neglected. More work is needed (e.g. constructing pavements) to support the high prevalence of walking reported in this study.

## Conclusions

Most children in Hyderabad walk (57 %) or cycle (6 %) to school. If these levels are to be maintained, there is an urgent need to ensure that walking and cycling are safe and pleasant. Social policies that decrease distances to school could have a large impact on road traffic injuries, air pollution, and physical activity levels.

## References

[1] Ministry of Statistics and Program Implementation: Ch 20 Motor Vehicles in India. 2015.

[2] Road Transport in India 2010-30 - Emissions, Pollution, and Health Impacts [May 2015]. Available from: http://urbanemissions.info/india-road-transport. Accessed Sept 2015.

[3] UNICEF. India: The children 2012 [Nov 2012]. Available from: http://www.unicef.org/india/children_166.htm. Accessed Sept 2015.

[4] Comprehensive Transportation Study of Hyderabad: Report on Development, Validation and Calibration of UTP Model, Scenarios and Travel Demand Forecast. 2012.

[5] National Household Travel Survey: Travel to School: The Distance Factor. Federal Highway Administration. 2008. http://nhts.ornl.gov/briefs/Travel%20To%20School.pdf.

[6] Dalton MA, Longacre MR, Drake KM, Gibson L, Adachi-Mejia AM, Swain K (2011). Built environment predictors of active travel to school among rural adolescents. Am J Prev Med.

[7] Silvia H. The Effect of School Quality and Residential Environment on Mode Choice of School Trips. TRB 2011 Annual Meeting; 2011

[8] Ziviani J, Scott J, Wadley D (2004). Walking to school: incidental physical activity in the daily occupations of Australian children. Occup Ther Int.

[9] Bringolf-Isler B, Grize L, Mader U, Ruch N, Sennhauser FH, Braun-Fahrlander C (2008). Personal and environmental factors associated with active commuting to school in Switzerland. Prev Med.

[10] NCSRTS. Safe Routes to School Travel Data: A Look at Baseline Results from Parent Surveys and Student Travel Tallies 2010. http://www.saferoutesinfo.org/sites/default/files/SRTS_baseline_data_report.pdf.

[11] Zhou HZJ, Hsu P, Rouse J (2009). Identifying Factors Affecting the Number of Students Walking or Biking to School. ITE Journal.

[12] Nelson NM, Woods CB (2010). Neighborhood perceptions and active commuting to school among adolescent boys and girls. J Phys Act Health.

[13] Panter JR, Jones AP, Van Sluijs EM, Griffin SJ (2010). Neighborhood, route, and school environments and children’s active commuting. Am J Prev Med.

[14] DiGuiseppi C, Roberts I, Li L, Allen D (1998). Determinants of car travel on daily journeys to school: cross sectional survey of primary school children. BMJ (Clinical research ed).

[15] Central Board of Secondary Education, India. Affiliation Bye-Laws, Chapter –I 1988. Available from: http://cbse.nic.in/affili~1/aff.pdf. Accessed Sept 2015.

[16] Guthold R, Cowan MJ, Autenrieth CS, Kann L, Riley LM (2010). Physical activity and sedentary behavior among schoolchildren: a 34-country comparison. J Pediatr.

[17] Tetali S, Edwards P, Murthy GV, Roberts I (2015). Development and validation of a self-administered questionnaire to estimate the distance and mode of children’s travel to school in urban India. BMC Med Res Methodol.

[18] State Education Department [cited 2014 04/05]. Available from: http://www.ap.gov.in/Other%20Docs/EDUCATION.pdf. Accessed Sept 2015.

[19] Educational statistics, Government of Andhra Pradesh. In: Commissioner& Director of School Education SPDRS, editor. 2011. http://mhrd.gov.in/sites/upload_files/mhrd/files/statistics/SSE1112.pdf.

[20] McDonald NC (2008). Children’s mode choice for the school trip : the role of distance and school location in walking to school. Transportation.

[21] McDonald NC, Brown AL, Marchetti LM, Pedroso MS (2011). U.S. School Travel 2009: An Assessment of Trends. Am J Prev Med.

[22] Panter JR, Jones AP, van Sluijs EM, Griffin SJ (2010). Attitudes, social support and environmental perceptions as predictors of active commuting behaviour in school children. J Epidemiol Community Health.

[23] O’Neill B, Mohan D (2002). Reducing motor vehicle crash deaths and injuries in newly motorising countries. BMJ (Clinical research ed).

[24] Lubans DR, Boreham CA, Kelly P, Foster CE (2011). The relationship between active travel to school and health-related fitness in children and adolescents: a systematic review. Int J Behav Nutr Phys Act.

[25] Mackett R, Brown B, Gong Y, Kitazawa K, Paskins J. Children’s independent movement in the local environment. Built Environ (1978-). 2007;33:454–68.

[26] M Badami GT, D Mohan. Access And Mobility For The Urban Poor In India: Bridging The Gap Between Policy And Needs. Forum on Urban Infrastructure and Public Service Delivery for the Urban Poor; 2004.

[27] Roberts I (2012). The truth about road traffic accidents. Br J Surg.

